# Opium

**Published:** 1878-03

**Authors:** 


### OPIUM.

The total amount of opium imported into the United States for 1877 was 2,588,924,383 grains. Deducting one-fifth for medical uses, there remains for opium eaters 6,125,-383 grains daily. If thirty grains are taken as a daily dose, there are in the United States over 200,000 persons who eat opium.

